# Digital Resilience-Based Course-Game Sequential Intervention for Social Participation in Stroke Survivors: Development and Usability Study

**DOI:** 10.2196/96250

**Published:** 2026-11-02

**Authors:** Xuan Zhou, You Lv, Runlin Liu, Wenjun Gao, Lanshu Zhou

**Affiliations:** 1 School of Nursing, Naval Medical University, 800 Xiangyin Road, Yangpu District, Shanghai, 200433, China, 1 13761801446; 2 Department of Hematology, The Second Medical Center & National Clinical Research Center for Geriatric Diseases, Chinese PLA General Hospital, Beijing, China

**Keywords:** stroke, social participation, intervention development, intervention mapping, course learning, serious game

## Abstract

**Background:**

Social participation is a key goal of stroke rehabilitation, yet effective interventions remain limited. Existing approaches focus largely on functional training or general psychological support, rarely targeting resilience as a modifiable mechanism for enhancing social participation. Most interventions are delivered face-to-face, limiting accessibility and scalability. A theory-driven digital intervention that integrates resilience training with contextualized practice may offer a promising pathway to support social reintegration in stroke survivors.

**Objective:**

This study aimed to develop a theory-driven, digital resilience-based course-game sequential intervention for improving social participation in stroke survivors and to evaluate its usability among the target population.

**Methods:**

This was an intervention development and usability study. Following the first 4 steps of intervention mapping, we completed the following steps: (1) conducted a needs assessment based on prior empirical evidence identifying psychological resilience as a key determinant of social participation; (2) defined performance and change objectives grounded in the Kumpfer resilience framework, which delineates 5 protective factors (cognitive, emotional, spiritual, behavioral, and physical); (3) selected theory-based methods and practical applications; and (4) developed an intervention prototype through 4 phases: initial design integrating course learning and gamified experiential modules, expert review and refinement by an 8-member panel, development of the digital system via a WeChat mini-program, and finalization of comprehensive intervention scripts. Participants were recruited using convenience sampling from a rehabilitation hospital in Shanghai, China. Usability was evaluated with 14 stroke survivors through 5 representative tasks, the System Usability Scale (SUS), and semistructured qualitative interviews. Quantitative data were analyzed using descriptive statistics, and qualitative data were analyzed using conventional content analysis.

**Results:**

The intervention comprised 2 sequential modules: a 7-session course learning module targeting 5 resilience factors, delivered weekly via narrative audio and multimodal content, and a gamified experiential module featuring a supermarket shopping scenario that enabled contextualized skill application. Each weekly course session was designed to last approximately 60 minutes. The duration of a single game session was 20 minutes. Participants had a mean age of 65.4 (SD 9.75) years, and 78.6% (11/14) had experienced an ischemic stroke. All participants completed the usability tasks successfully. The mean SUS score was 73.6 (SD 12.2, 95% CI 66.5-80.6; median 77.5, IQR 61.3-80.6), indicating good usability. Qualitative feedback revealed 3 themes: perceived ease of use and accessibility, perceived usefulness and engagement, and suggestions for improvement.

**Conclusions:**

This study systematically developed a theory-driven, digital course-game sequential intervention that is innovative in targeting psychological resilience as a core mechanism for social participation, distinguishing it from conventional function-focused or generic resilience approaches. By integrating systematic course learning with contextualized gamified practice, it offers a scalable, low-burden tool that addresses key gaps in stroke rehabilitation. These findings support progression to feasibility and efficacy testing through randomized controlled trials in real-world settings.

## Introduction

Social participation is increasingly recognized as the ultimate goal of stroke rehabilitation, a paradigm reinforced by the ICF (International Classification of Functioning, Disability and Health) [1]. The ICF conceptualizes participation as “involvement in a life situation,” though this definition remains broad and its operationalization has been subject to ongoing debate. Building on prior conceptual work, social participation has been further delineated to encompass several key features, such as engagement in social activities, fulfillment of social roles, and interaction with others in community settings [2,3]. However, stroke survivors frequently experience a significant and persistent decline in social participation [4,5], which is associated with elevated risks of low mood, recurrent stroke, diminished quality of life, and mortality [6-8]. Therefore, effective interventions to enhance social participation in this population are urgently needed.

Existing interventions aimed at improving social participation are diverse, including exercise-based programs, complex interventions, self-management strategies, and educational initiatives. Our recent scoping review and network meta-analysis revealed considerable heterogeneity in their effectiveness, with functional training remaining the dominant approach, while systematic interventions targeting psychological determinants are notably scarce [9]. This gap is significant, as negative affective states such as anxiety and depression have been consistently identified as potent barriers to poststroke social reintegration [10,11]. Moreover, the predominant face-to-face delivery mode is resource-intensive and may not be sustainable in settings with limited rehabilitation services, underscoring the need for more scalable digital approaches.

Psychological resilience, defined as the ability to adapt positively and “bounce back” in the face of adversity, offers a promising theoretical target. Based on the Kumpfer resilience framework, the initiation and maintenance of the resilience process are highly dependent on an individual’s internal protective traits, specifically cognitive, emotional, spiritual, behavioral, and physical domains [12,13]. Our previous research identified resilience as a crucial protective factor for social participation in stroke survivors, operating through a dual pathway: directly buffering psychological distress and indirectly enhancing participation by improving exercise adherence and subsequent physical function [14]. This finding suggests that the benefits of resilience extend beyond mere psychological well-being; they can catalyze positive behavioral changes, such as consistent exercise, that lead to better adaptive outcomes and, ultimately, greater community involvement.

Digital health interventions have emerged as effective tools for behavioral change in chronic disease management [15,16]. However, traditional mobile health applications in chronic care often focus primarily on the didactic provision of knowledge and skills, which can lead to user disengagement and high attrition rates due to a lack of interactivity [17,18]. Conversely, serious games, which are interactive computer applications designed for purposes beyond pure entertainment, such as training and education, have shown potential in chronic disease management [19-21]. Yet serious games face the inherent challenge of balancing entertainment with educational efficacy; excessive gamification may dilute the therapeutic message, while an overly serious approach can undermine user engagement [22]. To address these limitations, a synergistic approach that integrates the strengths of both conventional digital interventions and gamified components is warranted.

Therefore, grounded in the Kumpfer resilience framework and our prior work establishing psychological resilience as a key lever for improving social participation, this study aimed to develop a theory-driven digital intervention targeting the 5 core resilience factors through a sequential course-game design. The course learning module provides structured, accessible delivery of resilience skills, while the gamified experiential module offers immersive and contextualized practice, enabling participants to rehearse and reinforce these skills in a safe yet authentic environment. This paper describes the development of this digital, course-game sequential intervention using the intervention mapping (IM) approach and presents the results of its initial usability evaluation among stroke survivors.

## Methods

### Intervention Development Framework

IM is a systematic framework for developing, implementing, and evaluating behavior change interventions [23]. It comprises six consecutive steps: (1) conducting a needs assessment to identify health problems and their underlying determinants; (2) specifying performance objectives and their modifiable determinants; (3) selecting theory-based methods and practical strategies; (4) designing the intervention scope, sequence, and materials; (5) planning implementation; and (6) developing an evaluation plan. This study focused on the first 4 steps of IM, which guide intervention development. The implementation and evaluation of the intervention will be reported separately. This study adheres to the Guidance for Reporting Intervention Development [24].

### Step 1: Needs Assessment

A multidisciplinary team comprising physiotherapists, psychologists, nurses, and digital health experts was established to guide the intervention development. Building on our prior research identifying psychological resilience as a critical determinant of social participation in stroke survivors [14], the team adopted the Kumpfer resilience framework [12] as the core theoretical foundation. This framework delineates 5 interconnected protective factors that contribute to an individual’s capacity for resilience: cognitive competencies (rational thinking to counteract negative thought patterns); emotional management (identifying and regulating negative emotions); spiritual characteristics (belief in one’s ability to improve circumstances and achieve goals); behavioral competencies (problem-solving skills that enhance coping self-efficacy); and physical well-being (sound physiological foundations that facilitate positive psychological states). In this study, these 5 factors from the Kumpfer framework were operationalized as key modifiable determinants to guide the development of a targeted social participation intervention.

### Step 2: Behavioral Determinants and Performance Objectives

In this step, we first specified the overarching intervention goal: to enhance both the level and quality of social participation among stroke survivors. This goal was then delineated into five specific performance objectives: (1) formulating a personal social participation plan; (2) developing strategies for meaningful social engagement; (3) participating in self-selected social activities; (4) playing an active role within chosen social activities; and (5) experiencing a sense of fulfillment and positivity derived from social participation. Building on the needs assessment, these 5 determinants were linked to each performance objective through a matrix (Table S1 in Multimedia Appendix 1), specifying the targets for change required to achieve the intervention goal. For example, to address the emotional determinant, the matrix specified that patients need to reduce anxiety related to social participation and feel positive about formulating a participation plan, which directly supports the performance objective of “making plans for social participation.” A complete mapping of all 5 determinants to each performance objective is presented in Table S1 in Multimedia Appendix 1.

### Step 3: Theory-Based Methods and Practical Applications

Step 3 involved selecting appropriate theory-based and evidence-based methods to bridge the determinants with desired outcomes. Theoretical methods were defined as those derived from theory or research that aimed to modify determinants, while practical applications referred to specific strategies for implementing these methods [25]. The selection of theory-based methods was carried out by the group through three distinct approaches: (1) referencing the IM taxonomy of behavior change methods, (2) consulting the taxonomy of behavior change techniques (BCTs) [26], and (3) considering current empirical evidence. The team then discussed and identified feasible practical strategies to translate these theoretical methods into practice. Table S2 in Multimedia Appendix 1 provides a comprehensive overview of the theories and BCTs used to operationalize each determinant. For instance, to operationalize the emotional determinant, we selected BCTs related to emotion regulation (eg, BCTs-11.2) based on the IM taxonomy and translated them into concrete techniques such as mindfulness and deep breathing exercises.

### Step 4: Producing the Prototype Program

Based on the aforementioned steps, a prototype of the digital intervention was developed in 4 distinct phases.

#### Phase 1: Intervention Conceptualization and Design

The intervention prototype was designed to target both individual psychological characteristics and their precise application within specific social participation contexts. The proposed intervention consisted of 2 sequential components: a course learning module and a gamified experiential module. The course learning module provided contextualized training targeting the 5 resilience determinants. This included fostering positive cognitions regarding social participation, guiding the application of emotion regulation techniques to manage social interaction–related negative emotions, and assisting in the formulation of social participation goals and corresponding behavioral skills. Subsequently, the gamified experiential module simulated commonly encountered real-world barriers to social participation (eg, public skepticism and environmental obstacles). This design enabled individuals to naturally apply the skills acquired during the course learning module when confronted with real social situations, thereby facilitating the formation of efficient situation-response associations. The course learning and gamified experiential modules were thus designed as an integrated sequential system, with the former providing systematic learning and the latter offering a context for practical application. The finalized intervention protocol (Table 1) integrates the determinants, performance objectives, and practical strategies across all sessions, illustrating the complete logic chain from theoretical foundations to practical content. The mapping of determinants to objectives and methods is detailed in Tables S1 and S2 in Multimedia Appendix 1, respectively. A detailed mapping of each course learning session and game level to the corresponding ICF participation domains is provided in Table S3 in Multimedia Appendix 1.

**Table 1.** Overview of the digital resilience-based course-game sequential intervention sessions.

Module and week	Topic	Theoretical basis	Objectives	Content
Course learning				
1	Regulate emotions: stabilizing inner state for social participation	Emotional	To master general emotion regulation techniques and apply them to manage specific emotions arising in social participation contexts	General skills: Introduce changes brought by stroke and normalize poststroke emotional fluctuations Teach patients to identify negative emotions such as anxiety, frustration, and anger Teach patients emotion regulation techniques such as mindful breathing and emotional acceptance Contextual application: Encourage patients to use emotion regulation techniques to cope with anxiety triggered by social participation or fear of others’ evaluation Teach patients to handle frustration or anger arising from the inability to fulfill previous social roles Teach patients to calm feelings of frustration and impatience due to slow or fluctuating progress in social participation
2	Building positive cognitions for a new life chapter	Cognitive	To master general cognitive restructuring skills and apply them to establish positive beliefs about social participation	General skills: Guide patients to learn to identify and challenge negative automatic thoughts (eg, catastrophizing, all-or-nothing thinking, and overgeneralization) Cultivate patients’ ability to find exceptions and remaining strengths in adversity Contextual application: Encourage patients to reshape cognition regarding self-worth under functional limitations (eg, shifting focus from “instrumental value” to “existential value,” such as reframing “I cannot manage household chores for my family” to “I can express care verbally and become the family’s emotional pillar”) Encourage patients to reshape cognition regarding others’ evaluations Encourage patients to reshape cognition about social participation: correct erroneous beliefs, such as “Participation is only meaningful if I recover to my former state” or “Such minor participation doesn’t count as returning to society”
3	Finding meaning: cultivating motivation for community reintegration	Spiritual (motivational)	To discover inner values and meaning in life within the context of disability and connect them with social participation	General skills: Guide patients to reflect on core personal values under disability (eg, meaningful connections, helping others, and personal growth) Encourage patients to assign positive meaning to daily life events (eg, holding a cup steadily and walking 1 step further than yesterday) Guide patients to establish a personal mission that provides direction and purpose in daily life Contextual application: Help patients understand the significance of social participation and the consequences of nonparticipation Encourage patients to explore social activities that align with personal values (eg, “maintaining contact with old friends” fulfills the value of meaningful connection) Encourage patients to explore social roles they can still perform with their current abilities and provide stories of peers’ positive transformations
4	Managing energy: conserving physical and mental resources for engagement	Physical	To adhere to rehabilitation exercises and enhance the physical capacity required for social participation	General skills: Provide effective strategies for improving physical capacity (methods, timing, and strategies for home-based rehabilitation exercises) Share functional exercise methods used by other stroke patients Contextual application: Provide social participation techniques and compensatory strategies used by other patients Guide patients to establish social participation techniques and strategies suitable for their own abilities
5	Taking initiative: becoming the architect of your social life	Behavioral	To master general methods for goal decomposition and action planning and apply them to plan social participation behaviors	General skills: Teach goal-setting techniques Teach plan formulation techniques Teach problem-solving techniques Contextual application: Guide patients to set social participation goals based on self-selected activities Explain potential barriers and facilitators for poststroke social participation, guiding patients to identify factors relevant to themselves Guide patients to develop a social participation plan
6	Leveraging support: constructing a social network for participation	Behavioral	To develop the general ability to proactively build and use social support and apply it during the social participation process	General skills: Explain different types of social support (emotional, instrumental, informational, etc) Provide methods for making clear requests and expressing gratitude Explain how to proactively seek available resources and opportunities from the external environment Contextual application: Encourage patients to incorporate necessary social support into their social participation plans Encourage patients to communicate barriers to social participation with family members/relatives/friends and seek their help when necessary
7	Synthesis: narrating your resilience story in social participation	All elements	To integrate the learning journey into a coherent, positive narrative of personal resilience and growth, thereby consolidating changes	General skills: Guide patients to review and connect their progress in cognitive, emotional, spiritual, behavioral, and physical aspects Help patients summarize a resilience toolkit for coping with future social participation challenges Contextual application: Guide patients to share successful experiences and minor progress in social participation during the intervention period Help patients reinforce a positive self-identity of being prepared to face more social participation challenges Encourage patients to develop a flexible long-term plan for the next stage of social participation
Gamified experiential				
7 (postcourse)	Supermarket mission	All elements	To apply learned skills in a virtual social context and promote their transfer to real life	Within the supermarket shopping scenario, stroke survivors experience various obstacles that they may encounter from leaving home to checkout and use targeted strategies to address them: Cognitive: when facing psychological barriers like “fear of leaving home” or “negative evaluations from others,” guide players to identify and challenge catastrophic thinking, establishing positive beliefs through cognitive restructuring Emotional: when feeling embarrassed about “asking store staff for help” or “facing impatient others,” use emotion regulation techniques to manage social anxiety Physical: simulate real-world scenarios like “going up/down stairs” and “walking on roads” in the game. Guide patients to use crutches and negotiate steps using the appropriate movement sequence Spiritual: after completing the ultimate shopping challenge, reinforce the sense of achievement and meaning through system feedback

#### Phase 2: Expert Review and Protocol Refinement

An expert panel meeting was convened on October 17, 2025, to review and refine the preliminary intervention prototype. Eight experts were purposively selected according to the following criteria: (1) expertise in rehabilitation medicine, nursing, psychology, or cerebrovascular disease; (2) a senior professional title; (3) a master’s degree or higher; (4) at least 10 years of professional experience; (5) frequent interaction with stroke survivors; and (6) voluntary participation. The expert authority coefficient (Cr) was calculated as the mean of the familiarity coefficient (Cs) and the judgment coefficient (Ca), with a Cr value greater than 0.70 indicating a reliable opinion. No formal consensus threshold was predefined beyond this criterion, and the panel served as a formative expert review rather than a formal validation. Expert familiarity was assessed using a 5-point Likert scale, with scores assigned as follows: “very familiar” was assigned a score of 1.0, “familiar” a score of 0.8, “moderately familiar” a score of 0.6, “slightly familiar” a score of 0.4, and “not familiar” a score of 0.2. Expert judgment was based on 4 dimensions: practical experience, theoretical analysis, knowledge of current domestic and international evidence, and intuitive judgment. Each dimension was rated as “large,” “moderate,” or “small,” with specific scoring details provided in Table S4 in Multimedia Appendix 1. With the experts’ consent, the entire meeting was audio-recorded to capture their evaluations and feedback on the intervention protocol. The recordings were transcribed and analyzed promptly after the meeting. The transcribed feedback was reviewed and categorized thematically by the research team to identify key suggestions for refinement. The intervention protocol was subsequently revised based on the experts’ opinions, and disagreements were resolved through discussion and analysis among the research team members.

#### Phase 3: Digital System Development

Given its widespread use among older adults in China, a WeChat (Tencent Inc) mini-program was developed to deliver the intervention content. The course learning module transformed the content into a standardized, multimedia, interactive sequence, with users progressing by clicking “next” through text, images, videos, case examples, and interactive questions. To align with the intended “once-weekly” intervention dose, a weekly course unlocking mechanism was implemented, accompanied by scheduled push notifications reminding users to engage with the content. A dedicated “my social participation profile” module generated personalized reports upon course completion, allowing users to view and edit their participation elements and to receive visual progress feedback. The gamified experiential module featured a “supermarket shopping” scenario where users applied resilience skills to overcome simulated obstacles across 5 levels. An auxiliary resource library provided educational materials and self-assessment tools. The backend system enabled researchers to manage content and automatically recorded granular usage data, including course completion, learning duration, game performance, and interactive responses, providing an objective foundation for assessing adherence and informing future evaluations.

#### Phase 4: Content Finalization

Comprehensive intervention scripts were developed based on the refined intervention, culminating in a complete content repository for the course-game sequential intervention system. The final course learning module comprises approximately 50,000 characters, more than 140 images, 11 videos, more than 20 case examples, and more than 10 interactive questions. The gamified experiential module consists of 5 levels with distinct obstacles and problem-solving mechanisms, supported by approximately 6400 characters of script. This repository encompasses all materials necessary to deliver the theoretically grounded, digitally mediated intervention as designed.

### Usability Evaluation

#### Study Design

This was a formative usability evaluation of a digital intervention prototype.

#### Setting

Participants were recruited using a convenience sampling approach from a rehabilitation hospital in Shanghai, China, between October and November 2025.

#### Participants

The inclusion criteria were as follows: (1) diagnosis of stroke confirmed by the Fourth National Conference on Cerebrovascular Disease criteria [27]; (2) aged 18 to 80 years; (3) stable vital signs; (4) at least 1 month poststroke onset; (5) ability to use a smartphone and prior experience with WeChat; and (6) provision of written informed consent. Participants were excluded if they had the following: (1) cognitive impairment (a Montreal Cognitive Assessment score of less than 26; for participants with fewer than 12 years of education, 1 point was added to the raw score); (2) language dysfunction; (3) other serious medical conditions (eg, malignancy or organ failure); or (4) were illiterate.

#### Sample Size

The sample size of 14 falls within the recommended range for formative usability studies [28].

### Measures and Data Collection

Following the ISO 9241‐11 usability framework [29], five representative tasks were designed to evaluate the intervention’s usability: (1) locating and reading the “how to detect stroke early” section; (2) accessing and completing the “building positive cognitions for a new life chapter” learning module; (3) navigating to the game page and completing level 1; (4) viewing personal goals in the “my profile” section; and (5) completing a social participation self-test available in the “my profile” section. For each task, success was defined as reaching the designated interface element and completing the required action without external assistance. Task completion time was measured from the start of the task to its successful conclusion. To enable subsequent analysis, we recorded both the time taken to locate the target interface element and the total time to complete each task. To standardize administration, all tasks were presented to participants in the same fixed sequence.

After task completion, participants completed the System Usability Scale (SUS) [30], a 10-item questionnaire using a 5-point Likert scale (0=strongly disagree to 4=strongly agree). Odd-numbered items are positively worded, and even-numbered items are negatively worded. The SUS scores were calculated using the standard scoring procedure, yielding a total score ranging from 0 to 100, with higher scores indicating better usability. Scores above 60 indicate acceptable usability, with scores above 70 considered good, above 80 very good, and above 90 excellent [30]. The Chinese version of the SUS has demonstrated robust reliability and validity (Cronbach α=0.91) [31,32].

Semistructured individual interviews were subsequently conducted to explore participants’ perceptions and suggestions regarding the digital intervention. The interview guide included questions such as the following: “What was your overall experience with the digital system?”; “What are your thoughts on the font size and page color?”; “Do you find this digital system smooth to use?”; and “What shortcomings did you perceive in the digital system?” All interviews were audio-recorded and transcribed verbatim within 48 hours.

### Data Analysis

Quantitative data were analyzed using SPSS (version 27.0; IBM Corp). Descriptive statistics were calculated for all variables. Continuous variables were presented as means with SDs and 95% CIs, or as medians with IQRs, where appropriate. Categorical variables were reported as frequencies and percentages. Item-level SUS responses were also examined descriptively. Task completion times were reported as means with SDs. No missing data were present, so no imputation methods were applied.

Qualitative data from the semistructured interviews were analyzed using conventional content analysis [33]. The first author conducted and transcribed all interviews. The first author was a nursing researcher who was involved in the intervention development, and the second coder was a nursing researcher who was not involved in the intervention development. Two researchers independently coded the transcripts using conventional content analysis. Initial codes were identified from meaningful text units and then grouped into categories and synthesized into themes. Discrepancies were resolved through discussion, with a third researcher consulted if needed. Given the formative nature of this evaluation, no formal saturation criterion was applied, and member checking was not performed. The analysis was primarily descriptive, focusing on actionable feedback for intervention refinement.

### Ethical Considerations

This study was conducted in accordance with the Declaration of Helsinki and approved by the Ethics Committee of Shanghai First Rehabilitation Hospital (approval number: YK-2025-03-011). All participants provided written informed consent prior to enrollment. They were informed of the study purpose, procedures, potential risks, and of their right to withdraw at any time without consequence. To protect participant privacy, all data were deidentified and stored securely on password-protected computers accessible only to the research team. No participant compensation was provided for this usability evaluation. All images presented in this manuscript depict the intervention interface only and contain no identifiable information about any participant.

## Results

### Intervention Prototype Overview

The initial intervention protocol comprised 2 sequential components: a course learning module and a gamified experiential module. The course learning module constituted the core of the intervention system, consisting of 7 weekly thematic sessions. The seven themes were as follows: (1) building positive cognitions for a new life chapter; (2) regulating emotions: stabilizing inner state for social participation; (3) finding meaning: cultivating motivation for community reintegration; (4) managing energy: conserving physical and mental resources for engagement; (5) taking initiative: becoming the architect of your social life; (6) leveraging support: constructing a social network for participation; and (7) synthesis: narrating your resilience story in social participation. The content of the 7 sessions was connected through a coherent storyline and presented in a multimodal format, including integrated text, images, videos, case examples, and interactive questions.

The gamified experiential module was designed as a “supermarket shopping” journey scenario that gamified various obstacles encountered across different situations and guided users to apply resilience skills acquired during the course learning module to address these challenges. The game scenario was centered on a stroke survivor with moderate right-sided functional impairment who was preparing a birthday dinner for their spouse, encompassing 5 core levels: leaving home, the community park, the supermarket entrance, product selection, and supermarket checkout. These 5 levels were deliberately designed to correspond to the five resilience components. Specifically, leaving home addressed cognitive skills by challenging fears of negative evaluations from others. The community park and supermarket entrance targeted emotional and physical skills respectively, requiring emotion regulation for social anxiety and correct movement sequences for environmental navigation. Product selection engaged behavioral skills through goal-oriented decision-making. Supermarket checkout reinforced spiritual skills by providing achievement feedback upon task completion. This alignment is detailed in Table 1. The duration of a single game session was approximately 20 minutes.

### Expert Review and Protocol Refinement

A total of 8 experts were included in this study, with their demographic characteristics presented in Table S5 in Multimedia Appendix 1. The mean expert authority coefficient was 0.88 (SD 0.07; Table S6 in Multimedia Appendix 1). Overall, experts expressed broad recognition of the proposed resilience-based course-game sequential intervention for enhancing social participation in stroke survivors, acknowledging its theoretical coherence and integrated design. Nevertheless, specific suggestions for improvement were proposed, including recommendations regarding intervention dose, session order adjustments, content refinement, and increased interactive elements. Based on this expert feedback, the intervention protocol was appropriately modified and refined, as detailed in Table S7 in Multimedia Appendix 1. The finalized intervention protocol is presented in Table 1.

### Digital System Development

The intervention was implemented as a WeChat mini-program named “I Love Participating.” The name embodies the intervention’s core aim: to inspire and support patients in developing a passion for, and actively engaging in, social life. Representative screenshots of the intervention system are presented in Figure 1. To address the reading and cognitive challenges commonly faced by older adults, a narrative, audio-driven course model was developed. Users experience the intervention as if listening to a tailored, serialized radio drama, learning through a warm storyline via “audio guidance+multimodal formats” (texts, images, videos, and authentic interview cases). Furthermore, numerous first-hand cases obtained from our previously published qualitative study [34] were integrated to ensure the content directly addresses patients’ core psychological concerns. That study involved in-depth, semistructured interviews with 36 stroke survivors and explored their experiences of social participation, providing rich case materials for intervention script development [34]. Through audio guidance and minimal interaction, users can access practical resilience-building content in a relatively low-burden environment. The intervention is delivered as a self-guided program with a fixed content sequence. Technical support is available through a designated helpline and a WeChat contact for access-related issues; caregiver assistance is permitted but not required; missed sessions can be completed at any time after unlocking; adherence is defined as completion of all 7 course sessions and the game module; and fidelity is supported by the fixed content sequence and automated backend recording. A detailed script of approximately 50,000 characters was developed to ensure content consistency. Full operational details, including procedures for technical assistance, caregiver involvement, missed sessions, and fidelity criteria, are described in the published trial protocol [35].

**Figure 1.**
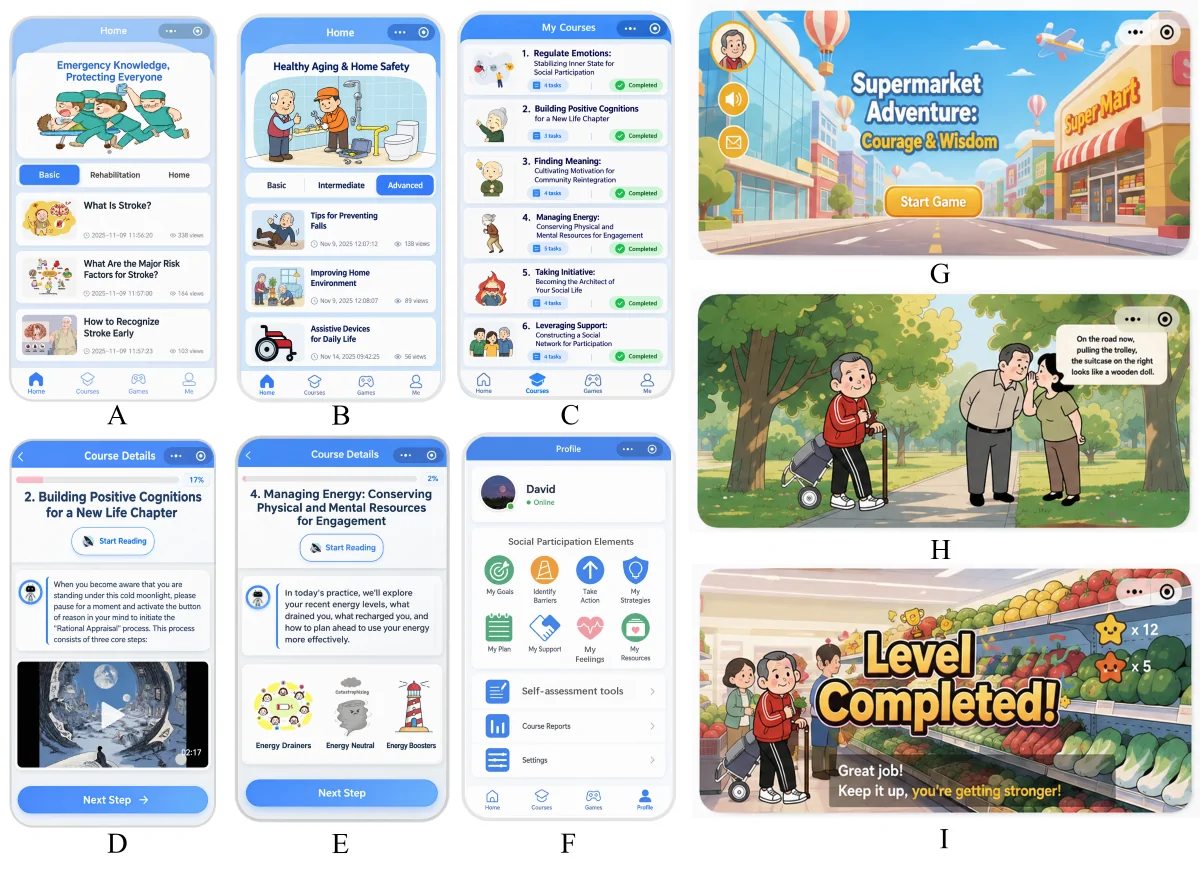
Representative interfaces of the digital resilience-based course-game sequential intervention. The WeChat mini-program consists of 4 main pages. The first page is the homepage, which presents stroke-related educational information (panels A and B). The second page is the course learning page, providing access to the 7 thematic course sessions (panel C), with each session delivered through a multimedia format integrating text, images, case examples, and videos (panels D and E). The third page is the gamified experiential module entry page, with the corresponding game interface shown in panels G, H, and I. The fourth page is the “my profile” section, which includes social participation elements and self-assessment tools (panel F).

### Usability Evaluation

#### Quantitative Findings

A total of 14 participants were included in the usability evaluation. The mean age of the participants was 65.4 (SD 9.75) years, with an age range of 45 to 80 years, and 71.4% (n=10) of the participants were male. Among the participants, 78.6% (n=11) had experienced an ischemic stroke. The duration from the initial stroke onset to study participation ranged from 1 month to 13 years. Participant characteristics are detailed in Table 2. The completion of representative tasks is documented in Table 3. Task 2 required completing an entire course topic with multimodal content, whereas the other tasks involved brief actions such as locating information, viewing personal goals, or completing a self-test. This difference in scope accounts for the large variation in completion times. All participants completed all 5 tasks successfully (14/14, 100% completion rate). However, because errors, assistance attempts, and first-click accuracy were not systematically recorded, this completion rate should not be interpreted as evidence of uniformly effortless or error-free interaction. The mean SUS score was 73.6 (SD 12.2), with a range of 55 to 95. The median score was 77.5 (IQR 61.3-80.6), and the 95% CI for the mean was 66.5 to 80.6. Item-level results, including the mean, SD, and median with IQR for each of the 10 SUS items, are presented in Table 4.

**Table 2.** The characteristics of the participants^a^.

ID	Sex	Age	Education	Employment status before stroke	Marriage status	Living status	Self-reported family economic status	Stroke type	Time since stroke onset	Recurrent stroke	Lateralization of motor impairment	Functional dependence level
1	Male	65	University	Retired	Married	Living with family	Very good	Ischemic	8 months	No	Left	Partly self-care
2	Female	45	Senior high school	Employed	Married	Living with family	Good	Ischemic	1 month	No	Left	Cannot take care of themselves
3	Male	67	Junior high school	Retired	Married	Living with family	Very good	Ischemic	2 months	No	Left	Mostly self-care
4	Male	69	Junior college	Retired	Married	Living with family	Average	Hemorrhagic	2 years	No	Right	Completely self-care
5	Male	58	Senior high school	Retired	Married	Living with family	Average	Ischemic	1 month	No	Right	Completely self-care
6	Male	80	Junior high school	Retired	Married	Living with family	Not good	Hemorrhagic	1 month	No	Left	Mostly self-care
7	Female	49	Senior high school	Employed	Married	Living with family	Average	Ischemic	1 month	No	Right	Partly self-care
8	Female	65	Senior high school	Retired	Married	Living with family	Average	Ischemic	1 month	No	Right	Mostly self-care
9	Male	70	Junior high school	Retired	Married	Living with family	Average	Ischemic	1 year	Yes	Both	Mostly self-care
10	Male	75	Junior college	Retired	Married	Living with family	Good	Ischemic	13 years	No	Left	Completely self-care
11	Male	65	Senior high school	Retired	Married	Living with family	Not good	Ischemic	9 years	Yes	Right	Partly self-care
12	Female	70	Primary school	Retired	Unmarried	Living alone	Good	Hemorrhagic	4 years	No	Both	Completely self-care
13	Male	61	University	Retired	Married	Living with family	Good	Ischemic	1 month	No	Left	Completely self-care
14	Male	76	University	Retired	Married	Living with family	Average	Ischemic	2 years	No	Left	Mostly self-care

a All variables were based on participant self-report.

**Table 3.** The completion of representative tasks (N=14).

Task	Completion rate (%)	Mean time to click on the target location (s)		Mean time to completion (min)	
		Mean (SD)	Median (IQR)	Mean (SD)	Median (IQR)
Task 1	100	16.92 (6.50)	15.60 (12.00-22.00)	2.82 (0.59)	2.95 (2.48-3.30)
Task 2	100	16.02 (7.12)	12.00 (8.00-16.00)	45.62 (5.46)	44.30 (42.00-48.65)
Task 3	100	12.80 (5.08)	15.00 (14.00-18.00)	5.12 (0.50)	5.05 (4.80-5.53)
Task 4	100	15.50 (4.83)	12.00 (12.00-19.00)	0.30 (0.05)	0.32 (0.27-0.34)
Task 5	100	11.51 (3.76)	10.00 (9.00-12.70)	8.73 (1.75)	8.50 (7.73-10.00)

**Table 4.** Item-level System Usability Scale (SUS) scores among stroke survivors (N=14).

Item	Description	Mean (SD)	Median (IQR)
1	Would like to use this system frequently	2.71 (1.07)	3.00 (2.00-3.25)
2	Found the system unnecessarily complex	1.00 (1.04)	1.00 (0.00-1.25)
3	Thought the system was easy to use	3.14 (0.54)	3.00 (3.00-3.25)
4	Would need technical support to use the system	1.14 (1.17)	1.00 (0.00-2.25)
5	Found various functions well integrated	2.79 (0.70)	3.00 (2.00-3.00)
6	Thought there was too much inconsistency	0.93 (0.73)	1.00 (0.75-1.00)
7	Most people would learn quickly	3.00 (1.04)	3.00 (2.75-4.00)
8	Found the system cumbersome to use	0.86 (0.86)	1.00 (0.00-2.00)
9	Felt confident using the system	3.07 (0.62)	3.00 (3.00-3.25)
10	Needed to learn a lot before getting started	1.36 (0.75)	1.50 (1.00-2.00)

#### Qualitative Findings

##### Theme 1: Perceived Ease of Use and Accessibility

Most participants found the interface intuitive and easy to navigate. Several participants specifically appreciated the audio-guided functionality, which they felt reduced the reading burden. One participant stated:

*I like the voice function. Since my illness, I have become more attentive to voice, which makes it easier for me to understand words.*

[Participant 1]

Another noted:

*The page color looks comfortable and refreshing.*

[Participant 3]

However, some participants expressed concerns about the font size, suggesting that it could be made adjustable to accommodate individual visual preferences.

##### Theme 2: Perceived Usefulness and Engagement

Participants generally viewed the intervention as potentially helpful for their social reintegration. Several expressed interest in the gamified experiential module, describing it as “novel and interesting” and noting that it enhanced their motivation to learn (Participant 6). The course reminder feature was also positively received, as 1 participant commented:

*When I get home, I may forget to use this application, and the reminder function is helpful.*

[Participant 3]

##### Theme 3: Suggestions for Improvement

Participants also provided constructive feedback for refinement. Regarding interaction design, some suggested incorporating more video-based content to enrich the learning experience and reduce the perceived monotony of text-heavy materials. Regarding content, a few participants expressed a desire for more concrete guidance on translating psychological resilience into actionable steps and connecting to broader support networks. Regarding intervention dose, while participants affirmed the value of the content and their motivation to learn, some expressed a preference for greater flexibility in the learning schedule rather than being restricted to unlocking one session per week. As one participant noted:

*I don’t think it’s necessary to limit me to one session per week. If I want to learn, I’m interested. Especially for those of us with hemiplegia, if I want to know more about this, I have the motivation. I don’t think it needs to be once a week.*

[Participant 8]

These suggestions indicate that allowing participants greater autonomy in pacing may enhance user experience and accommodate individual learning preferences.

## Discussion

### Principal Findings

This study systematically developed a theory-driven, digital course-game sequential intervention using the IM approach to support social participation among stroke survivors. Grounded in the Kumpfer resilience framework, the intervention targets 5 resilience-related protective factors through 2 sequential modules: a 7-session course learning component for systematic skill acquisition and a gamified experiential module, which features a “supermarket shopping” scenario for contextualized behavioral practice. The usability evaluation provided initial evidence of good usability among stroke survivors, supporting further efficacy evaluation.

Existing stroke rehabilitation has largely emphasized functional restoration [36,37], while psychological interventions have primarily focused on reducing negative affect, such as anxiety and depression [10,11]. Resilience, in contrast, has not been systematically operationalized as a core intervention target for promoting social participation. The first distinguishing feature of the intervention is its explicit use of psychological resilience as a theoretically specified mechanism for supporting social participation. Resilience is conceptualized as a dynamic process that enables individuals to adapt positively when confronted with adversity [38,39]. Our previous work further suggested that resilience may contribute to social participation through both direct psychological pathways and indirect behavioral pathways involving exercise adherence and physical function [13,14]. This evidence positions resilience as a higher-order determinant that can trigger a cascade of positive adaptive responses, making it a more potent target than addressing isolated psychological or functional deficits [40].

A second contribution is the contextual adaptation of established resilience-building strategies to stroke-specific social participation challenges. Previous resilience interventions may provide limited opportunities for context-specific practice. Although they commonly teach general coping and emotion-regulation strategies, translating these skills into demanding real-world social situations can remain challenging [41,42]. For instance, while these interventions commonly teach generic stress management techniques, they frequently fail to provide specialized training on how to initiate and apply these skills within concrete, demanding social situations [43]. This gap often results in a dilemma where participants understand the underlying principles yet struggle to implement them effectively when confronted with real-world social pressures [44,45]. The present intervention addresses this gap by transitioning from generic resilience education to contextualized resilience training. We focused not only on individuals’ intrinsic psychological traits but also anchored the intervention within specific social participation contexts. On the one hand, through the course learning module, we delivered contextualized training targeting the 5 resilience dimensions, including fostering positive cognitions regarding social participation, guiding the application of emotion regulation techniques to manage social interaction-related negative emotions, and assisting in setting social participation goals and formulating action plans. On the other hand, the gamified experiential module realistically simulates real-world barriers to social participation, enabling individuals to rehearse the acquired resilience skills when encountering simulated participation-related challenges [46].

A third contribution concerns the sequencing of structured digital learning and contextual game-based practice. Existing digital health interventions can efficiently provide health information, behavioral strategies, reminders, and self-management support, but predominantly didactic formats may be limited in their capacity to support experiential application and sustained engagement [15-18,47]. Conversely, serious games may promote active participation and motivation but can face tension between educational content and entertainment value [22,48]. The present intervention combines these complementary modalities within a learning-to-practice pathway: the course learning module provides structured acquisition of resilience-related knowledge and skills through narrative audio guidance and multimodal content, while the subsequent game enables contextualized rehearsal in a simulated social-participation scenario. The digital design was also adapted to the target population through delivery via a WeChat mini-program, audio narration, and simplified navigation to reduce reading and operational burden. Participants generally supported the accessibility of these features, particularly the audio-guided content, while suggesting further refinements such as adjustable font size, greater use of video, and more flexible learning schedules [49]. The usability findings, particularly the mean SUS score of 73.6 (SD 12.2), which exceeds the benchmark for digital health apps [50], provide initial evidence that this sequential format is usable in the target population. However, these findings reflect initial usability rather than evidence that the sequential architecture improves clinical outcomes; comparative effectiveness and sustained engagement remain to be evaluated.

This study has several limitations. First, due to resource constraints, the current intervention includes only one comprehensive gamified case rather than providing dedicated scenarios aligned with each of the 7 thematic sessions. Consequently, it cannot offer highly targeted simulation training for each specific resilience dimension, which partially weakens the closed loop of learning, practice, and reinforcement. An ideal model would pair each core thematic session with a corresponding gamified scenario to achieve seamless integration of skill acquisition and application, which is an important direction for future research [51]. Additionally, the game protagonist was designed with a fixed impairment profile, which may not directly reflect the experiences of all stroke survivors. Future iterations could consider incorporating multiple character profiles with varying laterality, severity, and assistive device use to better accommodate the diversity of the target population. Second, the gamified scenario was developed within a Chinese urban context, which may limit its direct transferability to other cultural or geographic settings. Although the supermarket shopping framework was chosen for its cross-cultural familiarity as a common community activity, specific details may require adaptation for different cultural environments in future implementations. Third, the sample was limited to cognitively intact, smartphone-proficient stroke survivors, limiting generalizability to those with severe conditions, advanced age, or low digital literacy [52]. For these more vulnerable populations, the “digital divide” challenge inherent in digital health interventions may be particularly pronounced. Although our audio-guided design was intended to alleviate the reading burden, the participants in this study were all volunteers with some degree of digital literacy, suggesting that the observed usability may reflect an optimistic bias. Future efforts to extend this intervention to older adults, individuals with low educational attainment, or those with severe disabilities will likely require further adaptation and validation of interface interaction modalities, such as substituting clicking with voice input or incorporating caregiver-assisted delivery models. Fourth, our usability evaluation was conducted through a single, task-based session. The SUS scores thus primarily reflect first-impression usability rather than sustained engagement over time. Participants did not experience the actual weekly, multiweek intervention delivery as intended in the full protocol. Therefore, while the current findings support initial usability, conclusions regarding long-term acceptability or sustained use cannot be drawn from this single exposure. Future studies incorporating repeated measures over the full intervention period are needed to evaluate sustained engagement and acceptance. In addition, errors, assistance attempts, and first-click accuracy were not systematically recorded, and the fixed task sequence may have introduced learning or order effects. Fifth, although the intervention encourages the use of social support, its core remains focused on the individual patient. The family and social environment, as critical determinants of social participation [5], were not systematically addressed within the scope of this individually oriented intervention. Finally, as the primary aim of this study was formative usability evaluation, behavioral or clinical outcome measures were not included.

### Conclusions

This study systematically developed and evaluated a theory-driven, digital course-game sequential intervention using the IM approach to support social participation in stroke survivors. Grounded in the Kumpfer resilience framework and informed by prior empirical evidence, the sequential course-game design addresses critical gaps by transitioning from generic resilience education to context-specific training anchored in real-world social scenarios. The usability findings provide initial support for the intervention prototype among stroke survivors. This work offers a scalable, theory-driven tool that has the potential to be integrated into routine stroke rehabilitation to facilitate community reintegration. However, further feasibility and efficacy testing in real-world settings is warranted.

## Supplementary material

10.2196/96250 Multimedia Appendix 1 Intervention development, expert evaluation, and intervention components.

## Abbreviations

BCT: behavior change technique
ICF: International Classification of Functioning, Disability and Health
IM: intervention mapping
SUS: System Usability Scale
