# Supplementary material for: Digital Resilience-Based Course-Game Sequential Intervention for Social Participation in Stroke Survivors: Development and Usability Study
Source: JMIR Serious Games. 2026 Nov 2;14:e96250. doi: 10.2196/96250 (PMC13727025; doi:10.2196/96250)
Supplement: Supplementary file 1 [file games-v14-e96250-s001.docx]

Table S1 Matrix of change for performance objectives

| Performance objectives | Cognitive | Emotional | Spiritual | Behavioral skills | Physical |
| --- | --- | --- | --- | --- | --- |
| Make plans for social participation | Develop positive perceptions of social participation  Recognize the importance of planning for successful participation | Reduce anxiety related to social participation  Feel positive about formulating a social participation plan | Believe in their ability to manage post-stroke social life  Feel confident in their capacity to plan for social participation | Articulate methods for developing a social participation plan  Discuss the plan with family members | Develop a feasible social participation plan based on their functional abilities  Allocate appropriate time and frequency for social activities |
| Work out compensatory strategies for social participation | Identify potential barriers to and facilitators of social participation Conceptualize feasible strategies to overcome obstacles | Reduce fear of potential barriers to social participation  Feel optimistic about potential facilitators | Believe in their ability to manage participation barriers  Feel confident in developing their own engagement strategies | Utilize facilitators to formulate strategies  Develop preliminary strategies to address barriers  Proactively seek information or assistance to refine strategies | Develop participation strategies appropriate to their personal circumstances  Maintain rehabilitation adherence to provide a physiological foundation for sustained participation |
| Participate in self-selected social activities | Describe the importance of self-selected activities for social participation Understand that "participation" is the first step toward success | Feel anticipation about participating in self-selected activities  Experience reduced tension regarding participation in chosen activities | Achieve participation in the targeted activity  Feel increased confidence about future social engagement | Successfully complete participation in the self-selected activity  Seek assistance when necessary to facilitate participation | Complete participation in the targeted activity according to their physical capabilities  Utilize assistive devices as needed to complete the activity |
| Play a positive role in meaningful activities | Observe and understand the needs or emotions of other participants  Recognize that their own contributions are valuable | Feel optimistic about the role they play in social activities  Express a sense of belonging and feeling needed within the activity | Experience a sense of self-worth during the activity  Reinforce the belief that they remain a valuable individual | Express their own opinions or suggestions during the activity  Provide small forms of assistance to others | Complete moderately challenging tasks appropriate to their physical condition |
| Experience the positive sense brought by participating in the society | Identify positive feelings associated with participation (e.g., satisfaction, sense of achievement) Attribute positive emotional experiences to their own social participation | Experience positive emotions such as enjoyment and satisfaction during participation | Perceive a sense of meaning in post-stroke social life  Feel confident and optimistic about future social engagement | Share positive experiences of social participation with others | Perceive the positive physical effects of positive emotions |

Table S2 Theory-based methods and practical applications to the intervention

| Determinants | Theory-based methods | Practical Applications |
| --- | --- | --- |
| Cognitive | Cognitive behavior therapy | Help patients reframe their perceptions of disability and accept changes in body image  Help patients develop a positive mindset toward post-stroke life  Help patients develop a positive mindset toward post-stroke social participation |
|  | Motivation enhancement | Empower patients about advantages of social participation and consequences of non-participation,  Help patients to recall the successful experiences of social participation  Help patients to feel the positive sense brought by social participation |
| Emotional | Regulate negative emotions (BCTs-11.2) | Help patients understand that emotional fluctuations after stroke are normal  Help patients identify negative emotions such as anxiety, frustration, and anger  Teach patients regulation techniques including mindfulness, deep breathing, and emotional acceptance |
| Spiritual | Motivational Enhancement Theory | Help patients understand the meaning of social participation and consequences of non-participation  Help patients recall successful past experiences of social participation  Help patients experience positive feelings derived from social participation |
|  | Self-Efficacy Theory | Help patients recall successful experiences in overcoming adversity  Share experiences and strategies of social participation from other stroke survivors  Enhance patients' confidence in their ability to participate socially |
| Behavioral skills | Goal setting (BCTs-1.1) | Help patients identify personally meaningful social activities  Encourage patients to clarify their goals for social participation |
|  | Make planning (BCTs-1.4) | Encourage patients to assess barriers to and facilitators of social participation  Help patients formulate a social participation plan |
|  | Problem solving (BCTs-1.2) | Share experiences and strategies of social participation from other stroke survivors  Encourage patients to identify methods and strategies for overcoming barriers  Encourage patients to discuss feasible methods and strategies with family members  Encourage patients to utilize available social support |
|  | As a role model（BCTs-13.1） | Encourage patients to share their own social participation experiences and feelings with other stroke survivors |
|  | Self-monitoring of the behavior (BCTs-2.3 and 2.4) | Encourage patients to regularly evaluate their social participation status  Encourage patients to regularly revise their social participation plan |
|  | Feedback (BCTs-2.7) | Encourage family members to provide feedback on patients’ social participation behaviors |
|  | Social support (BCTs-3.1)  Social support theory | Encourage patients to utilize available social support  Encourage patients to seek assistance from family members when necessary  Encourage patients to formulate social participation plans and make decisions together with family members |
| Physical | Strengths-Based Perspective | Help patients recognize and appreciate the value inherent in their current physical abilities  Encourage patients to adhere to rehabilitation to enhance and maintain physical function |

Table S3 Mapping of intervention components to ICF participation domains

| Intervention component | Contextual application | ICF participation domain | Rationale |
| --- | --- | --- | --- |
| Regulating Emotions: Stabilizing Inner State for Social Participation | Encourage patients to use emotion regulation techniques to cope with anxiety triggered by social participation or fear of others’ evaluation.  Teach patients to handle frustration or anger arising from the inability to fulfill previous social roles.  Teach patients to calm feelings of frustration and impatience due to slow or fluctuating progress in social participation. | d7 Interpersonal interactions and relationships; d9 Community, social and civic life | Emotion regulation enables individuals to manage social anxiety and emotional responses in interpersonal and community settings |
| Building Positive Cognitions for a New Life Chapter | Encourage patients to reshape cognition regarding self-worth under functional limitations (e.g., shifting focus from “instrumental value” to “existential value,” such as reframing “I cannot manage household chores for my family” to “I can express care verbally and become the family’s emotional pillar”).  Encourage patients to reshape cognition regarding others’ evaluations.  Encourage patients to reshape cognition about social participation: Correct erroneous beliefs, such as “Participation is only meaningful if I recover to my former state” or “Such minor participation doesn’t count as returning to society.” | d7 Interpersonal interactions and relationships; d9 Community, social and civic life | Cognitive restructuring addresses maladaptive beliefs about self and others that impede social engagement |
| Finding Meaning: Cultivating Motivation for Community Reintegration | Help patients understand the significance of social participation and the consequences of non-participation.  Encourage patients to explore social activities that align with personal values (e.g., “maintaining contact with old friends” fulfills the value of meaningful connection).  Encourage patients to explore social roles they can still perform with their current abilities and provide stories of peers’ positive transformations. | d7 Interpersonal interactions and relationships; d9 Community, social and civic life | Discovering meaning and purpose motivates engagement in valued social activities and roles |
| Managing Energy: Conserving Physical and Mental Resources for Engagement | Provide social participation techniques and compensatory strategies used by other patients.  Guide patients to establish social participation techniques and strategies suitable for their own abilities. | d4 Mobility; d5 Self-care; d7 Interpersonal interactions and relationships; d8 Major life areas; d9 Community, social and civic life | Energy management supports functional capacity for mobility and self-care, while compensatory strategies enable continued participation across interpersonal, domestic, and community life domains |
| Taking Initiative: Becoming the Architect of Your Social Life | Guide patients to set social participation goals based on self-selected activities.  Explain potential barriers and facilitators for post-stroke social participation, guiding patients to identify factors relevant to themselves.  Guide patients to develop a social participation plan. | d4 Mobility; d5 Self-care; d6 Domestic life; d7 Interpersonal interactions and relationships; d8 Major life areas; d9 Community, social and civic life | This session equips participants with goal-setting and planning skills applicable across all participation domains, as reflected in the diverse activity examples provided in the course (work, family care, friendships, hobbies, community activities) |
| Leveraging Support: Constructing a Social Network for Participation | Encourage patients to incorporate necessary social support into their social participation plans.  Encourage patients to communicate barriers to social participation with family members/relatives/friends and seek their help when necessary. | d4 Mobility; d5 Self-care; d7 Interpersonal interactions and relationships; d8 Major life areas; d9 Community, social and civic life | This session teaches participants to mobilize social support across multiple life domains, as illustrated by case examples involving community mobility, employment, and use of community resources |
| Synthesis: Narrating Your Resilience Story in Social Participation | Sharing successful experiences and progress; reinforcing positive self-identity; developing a flexible long-term plan | d4 Mobility; d7 Interpersonal interactions; d8 Major life areas; d9 Community, social and civic life | Integration of resilience skills consolidates capacity for sustained participation across multiple life domains |
| Game Level 1: Leaving Home | Overcoming fear of leaving home and negative evaluations from others | d4 Mobility; d9 Community, social and civic life | Overcoming psychological barriers to mobility enables community access |
| Game Level 2: Community Park | Navigating public spaces and managing social anxiety in community settings | d4 Mobility; d7 Interpersonal interactions and relationships; d9 Community, social and civic life | Community navigation involves both physical mobility and social interaction |
| Game Level 3: Supermarket Entrance | Entering a public commercial space; managing environmental obstacles | d4 Mobility; d5 Self-care; d9 Community, social and civic life | Entering public spaces requires mobility, self-care, and community participation |
| Game Level 4: Product Selection | Making decisions in a public context; goal-oriented decision making | d2 General tasks and demands; d8 Major life areas; d9 Community, social and civic life | Decision-making in daily activities reflects general task demands and major life areas |
| Game Level 5: Checkout | Interacting with store staff; asking for help; completing a transaction | d7 Interpersonal interactions and relationships; d9 Community, social and civic life | Completing a purchase involves interpersonal interaction and community participation |

Table S4 Expert judgment basis and corresponding scores

| Judgment basis | Degree of influence as the basis for judgment | | |
| --- | --- | --- | --- |
|  | large | moderate | small |
| Theoretical analysis | 0.3 | 0.2 | 0.1 |
| Practical experience | 0.5 | 0.4 | 0.3 |
| Knowledge of current research status both domestically and internationally | 0.1 | 0.1 | 0.05 |
| Intuitive judgment | 0.1 | 0.1 | 0.05 |

Table S5 General Information of Expert Panel

| No. | Gender | Age | Professional title | Education | Academic Advisor | Area of Expertise | Years of Experience |
| --- | --- | --- | --- | --- | --- | --- | --- |
| 1 | Female | 55 | Senior | PhD | Doctoral Supervisor | Stroke Rehabilitation | 30 |
| 2 | Male | 51 | Senior | PhD | Doctoral Supervisor | Information Technology | 25 |
| 3 | Male | 50 | Senior | Master's | Master’s Supervisor | Stroke Rehabilitation | 30 |
| 4 | Female | 43 | Senior | PhD | Master’s Supervisor | Chronic Disease Management | 16 |
| 5 | Female | 42 | Associate Senior | PhD | Master’s Supervisor | Applied Psychology | 17 |
| 6 | Female | 44 | Associate Senior | PhD | Master’s Supervisor | Nursing Psychology | 15 |
| 7 | Female | 52 | Senior | Master’s | No | Rehabilitation Nursing | 11 |
| 8 | Male | 37 | Associate Senior | Master’s | No | Information Technology | 14 |

Table S6 Expert Authority Coefficients

| No. | Theoretical Analysis | Practical Experience | Reference to Literature | Intuition | Judgment Coefficient (Ca) | Familiarity Coefficient (Cs) | Authority Coefficient (Cr) |
| --- | --- | --- | --- | --- | --- | --- | --- |
| 1 | 0.30 | 0.50 | 0.10 | 0.05 | 0.95 | 1.00 | 0.98 |
| 2 | 0.20 | 0.50 | 0.10 | 0.10 | 0.90 | 0.80 | 0.85 |
| 3 | 0.30 | 0.40 | 0.10 | 0.10 | 0.90 | 0.80 | 0.85 |
| 4 | 0.30 | 0.40 | 0.10 | 0.05 | 0.85 | 0.80 | 0.83 |
| 5 | 0.30 | 0.50 | 0.10 | 0.05 | 0.95 | 0.80 | 0.88 |
| 6 | 0.30 | 0.50 | 0.10 | 0.05 | 0.95 | 0.80 | 0.88 |
| 7 | 0.30 | 0.50 | 0.10 | 0.05 | 0.95 | 1.00 | 0.98 |
| 8 | 0.20 | 0.40 | 0.10 | 0.10 | 0.80 | 0.80 | 0.80 |

Table S7 Expert Feedback and Corresponding Modifications

| Expert Feedback | | Response to Expert Feedback |
| --- | --- | --- |
| Intervention Dose | Expert 5: The 60-minute duration per session is too long; consider shortening it, for example to 10 minutes.  Expert 6: The 60-minute learning duration per session is somewhat lengthy; consider allowing the content to be completed in multiple sessions throughout the week.  Expert 8: Consider breaking down the 60-minute course into smaller segments. | The expert opinion was accepted. To maintain content integrity, each thematic session was designed to contain approximately 60 minutes of course content, representing the weekly learning requirement. However, patients are permitted to personalize their learning mode by dividing the content into multiple learning sessions and revisiting content of interest as needed. |
| Session Order | Expert 5: Consider adjusting the order of the first and second sessions, prioritizing emotional content before cognitive content. | The expert opinion was accepted. The order of Theme 1 and Theme 2 was adjusted accordingly. |
| Intervention Content | Expert 4: Regarding the operationalization of scenario-based applications, consider incorporating more interactive elements to enhance participants' engagement and adherence.  Expert 8: It would be beneficial to present interactions more clearly and avoid lengthy textual passages. | The expert opinion was accepted. When developing course lesson plans based on intervention themes and core content, efforts were made to segment the content appropriately and employ diverse presentation formats to ensure information clarity and highlight key points. |
|  | Expert 6: After completing one session per week, should homework assignments be considered? | While homework assignments can facilitate skill consolidation and generalization, to ensure intervention dose consistency and maximize adherence, formal mandatory homework assignments were not incorporated into the current protocol. |
| Relationship Between Course and Gamified Experiential Modules | Expert 1: Could the game be integrated with the course, allowing corresponding training after learning each thematic session?  Expert 8: Consider integrating the game components across the seven course sessions. | The “sequential” nature of this protocol specifically refers to completing systematic knowledge construction across all seven course sessions before engaging in a comprehensive game-based journey for overall application and skill integration. This design aims to ensure theoretical knowledge integrity and assess participants' ability to utilize multidimensional skills in integrated contexts. This approach serves as the foundation for validating the overall effectiveness of the protocol in the current study. The model proposed by experts, featuring “one session, one practice,” represents a valuable direction for comparative investigation in future research. |
| Delivery System | Expert 5: Consider developing an adaptive system that delivers personalized interventions based on individual patient needs. | The core theoretical construct of this intervention is based on Kumpfer’s Resilience Framework, which emphasizes that the five dimensions—cognitive, emotional, spiritual, behavioral, and physical—constitute an interconnected, holistic psychological structure. The primary objective of this study is to validate the overall effectiveness of this complete, structured sequential intervention protocol, rather than examining the independent effects of individual dimensions or conducting component adaptability testing. Employing a standardized intervention approach enables clear attribution of effects to the overall protocol. |
| Gamified Experiential Module | Expert 4: The current scenario features one case (a patient with moderate right-sided functional impairment). Consider incorporating greater character diversity, such as different impairment locations and severity levels. | The expert opinion is strongly endorsed. The initial design aspiration for the gamified scenarios was to match game cases based on data captured from the course learning module according to participant characteristics, such as functional severity, social participation goals, and assistive device use. However, this would involve diverse character features, various gamified scenarios, and each combination variations results in different game mechanics, leading to exponentially increased costs. Therefore, given current economic and time constraints, only one gamified scenario was developed. Future work will consider developing a gamified scenario repository and updating the gamified experiential module. |
|  | Expert 3: The design is excellent, and the game levels are representative. | The expert’s positive feedback is gratefully acknowledged. |
| Backend Data Recording | Expert 6: Consider backend recording of data on patient engagement, such as number of gameplay sessions or learning sessions. | The expert opinion was accepted. Data tracking points were embedded in the WeChat mini-program backend to record participant learning process data. This includes: (1) course learning module data, such as course completion rates and page dwell time; and (2) gamified simulation data, such as number of game attempts and virtual reward acquisition. |
| Future Application | Expert 2: It is hoped that this resilience course can be implemented as routine practice, administered upon hospital admission rather than only when problems emerge. | The expert opinion is strongly endorsed, and this direction aligns with the team's ongoing efforts. Preliminary research findings indicate that resilience not only directly influences social participation levels in stroke survivors with physical impairments but also indirectly enhances social participation through the sequential mediating pathway of improving rehabilitation exercise adherence and physical function. Previous qualitative research also revealed that stroke survivors with higher resilience levels demonstrate greater resilience in coping with physical impairments, maintain more optimistic emotions, and adopt more positive coping strategies. Through developing this theoretically grounded course-game sequential intervention aimed at enhancing social participation in stroke survivors with physical impairments, and if its effectiveness is validated through randomized controlled trials, it is hoped that this intervention can be integrated into routine care in stroke rehabilitation facilities, ultimately facilitating better community reintegration for stroke survivors. |
| Outcome Indicators | Expert 2: Outcome measures should encompass not only willingness to go outdoors but also online social engagement. | The primary outcome of this intervention protocol is assessed using the Utrecht Scale for Evaluation of Rehabilitation-Participation (USER-P). Additionally, qualitative interviews will be conducted at post-intervention time points to gather richer data on participants' social participation experiences. |
